# Evaluating the Appropriateness of Podcasts to Improve the Knowledge and Awareness of Selected Health Topics Among Undergraduate General Nursing Students: Protocol for an International Feasibility Study

**DOI:** 10.2196/50735

**Published:** 2024-02-06

**Authors:** Alanna Denny, Brian Curtin, Simon Taylor-Robinson, Griphin Baxter Chirambo, Liezel Cilliers, Tsung-Shu Joseph Wu, Ciara O'Meara, Richard Booth, John O'Donoghue

**Affiliations:** 1 University College Cork Cork Ireland; 2 Imperial College London London United Kingdom; 3 Faculty of Health Sciences Mzuzu University Mzuzu Malawi; 4 University of Fort Hare Alice Eastern Cape South Africa; 5 Research Department Luke International Mzuzu City Malawi; 6 Overseas Services Department Pingtung Christian Hospital Pingtung County Taiwan; 7 School of Nursing & Midwifery University of Galway Galway Ireland; 8 Western University Ontario, ON Canada; 9 ASSERT Research Centre University College Cork Cork Ireland; 10 Business Information Systems University College Cork Cork Ireland; 11 Malawi eHealth Research Centre Mzuzu University Mzuzu City Malawi

**Keywords:** podcasting, podcast, nursing student, gestational diabetes, mental health, health, knowledge

## Abstract

**Background:**

Podcasts have proven to be a successful alternative source of educational material for students. Given the ability to listen to podcasts 24/7 and while on the go, this technology has the potential to provide informative and educational material to a large number of people at any given time. Podcasts are usually freely available on commonly used mobile devices, such as smartphones, laptops, and tablets.

**Objective:**

This paper describes the impact of health-related podcasts as an intervention tool to support the knowledge and awareness of nursing students on a given topic.

**Methods:**

Pre- and postpodcast questionnaires will gather data regarding the participants’ knowledge and awareness of two topics—gestational diabetes and mental health. This intervention will be tested on general nursing undergraduate students. The total number of students (N=2395) from the participating universities are broken down as follows: (1) University College Cork (n=850) and the University of Galway (n=450) in Ireland, (2) Mzuzu University in Malawi (n=719), and (3) University of Fort Hare in South Africa (n=376).

**Results:**

The study received ethical approval from the University College Cork Ethics Committee (2022-027A1). The approval obtained from University College Cork sufficed as ethics coverage for the University of Galway in Ireland. Ethics approval was also received from the Mzuzu University Research Ethics Committee (ID MZUNIREC/DOR/23/28) and the Inter-Faculty Research Ethics Committee of the University of Fort Hare (ID CIL002-21). Data collection is currently underway and will continue until the end of February 2024. The quantitative and qualitative data are expected to be analyzed in March 2024.

**Conclusions:**

Results from this study will allow for an investigation into the impact of podcasts in different settings: a high-income country (Ireland), an upper-middle–income country (South Africa), and a low-to-middle–income country (Malawi). The data gathered from this feasibility study will provide more clarity on the potential utility of podcasts as an intervention tool. We will gather data regarding listener demographics (eg, country of residence, age, gender, and year of study).

**International Registered Report Identifier (IRRID):**

DERR1-10.2196/50735

## Introduction

### Overview

The term “podcast” has been used since approximately 2004. It originally stemmed from 2 terms “iPod” and “broadcast” [1]. The groundbreaking iPod device from Apple pioneered the mobility of digital audio information. Although the iPod has never been the only device used for podcast listening, it swiftly grew into one of the most prominent listening devices [2]. A podcast can be listened to on any digital audio listening device, including a computer that supports audio file playing. Therefore, podcasts can be listened to 24/7 and while on the go. These combined attributes have dramatically increased the use of podcasts over the last 20 years [3]. During this period, podcasts have been regarded as effective medical educational instruments [4,5]. Thus, the accessibility and open access of podcasts may be hugely attractive to higher education institutes as a method of teaching and learning.

Chin et al [6] (2017) showed that after listening to the extracurricular audio podcasts on health-related topics, students who completed the study experienced a significant and probably educationally relevant improvement in their knowledge. This aligns with the results of Mitchell et al [7] (2021), which showed an overall increase of 11.54% in knowledge attainment in undergraduate nursing students after listening to a podcast on delirium [7]. Overall, these studies demonstrate the impact of podcasting on knowledge attainment in third-level education.

A study of the literature in 2017 further highlights that podcasts are a popular source of educational material for a wide range of health care professionals, including junior doctors, nurses, and medical students [4]. In particular, third-level nursing education is an area of health care that calls for sophisticated critical thinking abilities and decision-making processes, which may likely influence clinical competency. University staff members can find themselves searching for new and creative ways to present material in an engaging fashion as the demands on their time and the availability of innovative tools for use in nursing education expand. The implementation of podcasting in nursing education is one strategy to enhance learning, as discussed in the literature. Recent research conducted in Northern Ireland on podcasting to improve undergraduate nursing students’ awareness of Parkinson Disease found that “podcasting as an educational resource for undergraduate student nurses is a successful way of developing knowledge for PD [Parkinson disease]” [8].

There has been a rise in research being conducted on podcasting in nursing education [9]. Despite this, there is limited research being undertaken on the use of podcasts in Africa, even though podcasting is a great topic of conversation among interested African communities. Surveys completed by the Reuters Digital News Report that included 2 African countries highlighted that 33% of participants were considered regular podcast users [10]. Notably, 50% believed that podcasts are more informative in terms of providing a better understanding of issues compared to traditional media platforms [10].

This research will allow for an investigation into the impact of podcasts on knowledge and awareness in different settings—a high-income country (Ireland), an upper-middle–income country (South Africa), and a low-to-middle–income country (Malawi).

Knowledge and awareness have often been used synonymously, but in this study, a distinction between these 2 interchangeable words is set. Knowledge has been described as “the fact or condition of knowing something with familiarity gained through experience or association,” the “acquaintance with or understanding of a science, art, or technique,” and “the range of one's information or understanding” [11]. Awareness has been described as “knowledge and understanding that something is happening or exists” [12]. In our study, awareness will be defined as having heard of a term or topic, while knowledge will be defined as a deeper understanding of the area.

The podcast topics are as follows:

#### Pregnancy

According to the World Health Organization (WHO), approximately 800 women died every day from pregnancy and childbirth-related avoidable causes in 2020 [13]. Of note, nearly 95% of these maternal deaths took place in low- and low-to-middle–income countries, such as Malawi [13]. This highlights the need to raise awareness of pregnancy risk factors, complications, and services available for women to access the care they need.

#### Gestational Diabetes

The WHO describes gestational diabetes as “hyperglycemia with blood glucose values above normal but below those diagnostic of diabetes,” which occurs during pregnancy [14]. Worldwide, 21.3 million pregnancies are linked to hyperglycemia, and of these, 18.4 million pregnancies are linked to gestational diabetes mellitus [15]. Furthermore, it is estimated that more than 90% of hyperglycemia cases in pregnancy occur in low- and middle-income countries [16]. Gestational diabetes can impact both the fetus and the mother. Long-term implications for children include an increased risk of obesity and metabolic disruption [17]. Furthermore, women who experience gestational diabetes are at risk for developing type 2 diabetes later in life [17]. This demonstrates that gestational diabetes a potentially preventable condition can have a huge societal burden and devastating long-term outcomes.

#### HIV/AIDS

The WHO figures highlight how HIV infection remains a significant global health concern with 40.1 million deaths occurring to date [18]. Approximately, 650,000 persons died in 2021 as a result of HIV-related causes, and by the end of 2021, an estimated 38.4 million people were living with HIV [18]. Nearly 60% of new HIV infections worldwide occur in the WHO African Region [18]. The HIV infection is incurable. However, owing to accessibility to effective HIV prevention, diagnosis, treatment, and care, especially for opportunistic infections, HIV infection has evolved into a manageable chronic health disease, allowing those who have it to live long and healthy lives. Unfortunately, the disease continues to take many lives despite the availability of treatment and hence it is an important podcast topic to discuss to increase knowledge and awareness.

#### Cervical Cancer

According to global estimates, there were 604,000 new cases and 342,000 deaths from cervical cancer in women worldwide in 2020 [19]. New cases of cervical cancer and deaths from cervical cancer that occurred in low- and middle-income countries accounted for around 90% of the global total deaths in 2020 [20]. Nearly 50% of high-grade cervical precancers are caused by 2 human papillomavirus types (16 and 18) [20]. This is an important topic to discuss as cervical cancer is curable if detected early and treated effectively. Preventing cervical cancer is more affordable with the human papillomavirus vaccine, precancer lesions screening, and treatment.

#### Malnutrition

The WHO defines malnutrition as “undernutrition (wasting, stunting, underweight), inadequate vitamins or minerals, overweight, obesity, and resulting diet-related noncommunicable diseases” [21]. A total of 462 million adults are underweight, compared to 1.9 billion who are overweight or obese [21]. Undernutrition is a contributing factor in about 45% of deaths in children younger than 5 years and mostly takes place in low- and middle-income countries [21]. The prevalence of childhood obesity and overweight is also rising in these same nations. The global burden of malnutrition has substantial and long-lasting effects on people and their families as well as on communities, nations, and the economy; therefore, greater conversation around this area is imperative.

#### Mental Health

As evidenced by the inclusion of mental health in the United Nations Sustainable Development Goals [22], there has been growing recognition of the crucial role mental health plays in achieving global development goals in recent years [23]. According to Arias et al [24] (2022), 418 million disability-adjusted life years or 16% of all disability-adjusted life years worldwide could be attributed to mental diseases in 2019. Overall, this confirms that the need for action on mental health topics is paramount.

### Objective of the Feasibility Study

This paper explores the impact of health-related podcasts as an intervention tool to support the knowledge and awareness of nursing students on a given topic. To address this objective, we aim to answer the following questions:

1. Can podcasts support listeners' knowledge of global health topics?
2. Is knowledge attainment from podcasts different across Ireland, South Africa, and Malawi?
3. Is podcasting effective for supporting awareness in nursing students on global health diseases and issues?

## Methods

### Research Design

This pre- and postpodcast intervention is a feasibility study. Feasibility studies facilitate researchers’ evaluation of whether an intervention is suitable to undergo additional investigation or whether the concepts and conclusions can be applicable and sustainable to the population [25]. A feasibility method was chosen for this study due to the lack of previously published studies on this specific intervention.

### Type of Study and Setting

This study mainly involves quantitative data, but qualitative data will be synthesized for the 1 open-ended question on the survey tool. These questionnaires will be created and managed using Google Forms, as it is compliant with the General Data Protection Regulation. Microsoft Excel will be used to capture qualitative and quantitative data and to execute descriptive statistics in the form of percentages and frequencies [26]. This study will be carried out from March 2023 until March 2024.

The selected podcast topics revolve around global health concerns that exhibit significant variations in understanding and practices across different countries. This is intended to enable in-depth podcast discussions and survey questions that can yield insights into how nursing students use the podcasts. These podcast topics have been chosen to encompass a diversity of health-related topics affecting both high- and low-income countries.

To date, the recordings of the podcasts have been conducted. Video and audio files for 2 out of the 6 podcasts will be available for the study participants. Despite only 2 podcasts being chosen for research purposes, all 6 podcasts will be disseminated on platforms including but not limited to YouTube and Spotify. The podcasts’ duration ranges from approximately 30 to 60 minutes each. Each podcast discusses the risk factors, symptoms, and treatment or management of the global health topic.

Each podcast was created with a panel of international experts along with a chair (AD and BC). The 2 podcasts selected for the study are the gestational diabetes podcast and the mental health podcast. As this is a voluntary feasibility project, we only have the resources to examine 2 of the podcasts. Upon completion of this research, we will determine if podcasting is an effective method of increasing knowledge and awareness attainment. If the results are positive, we will conduct a broader survey, which will include members of the public and not just nursing students, and will release more podcasts. In addition, mental health and gestational diabetes are widespread issues normally taught in depth in nursing schools and are not confined by geographical locations or socioeconomic status. Likewise, these podcasts had the best audio quality, conversation flow, and in-depth discussion, as decided by the research team. It is evident from the literature that gestational diabetes is a global phenomenon that can impact people from Ireland, South Africa, and Malawi and have potentially long-term preventable implications [16,17]. Similarly, mental health is a global health concern that is gaining increasing attention due to the addition of mental health in the Sustainable Development Goals and the disability-adjusted life years that are attributable to mental disease [23,24]. The selected global health topics are on the list of the top 10 global public health challenges to track in 2023 [27].

### Measurement Tools

For the gestational diabetes podcast, a 5-item “Yes-No-I don’t know” awareness questionnaire and a 27-item “Yes-No-I don’t know” knowledge questionnaire were developed by the researchers. For the mental health podcast, a 4-item “Yes-No-I don’t Know” awareness questionnaire and a 26-item “Yes-No-I don’t know” knowledge questionnaire were developed by the researchers. The gestational diabetes podcast has 1 additional survey question on awareness and knowledge compared to the mental health podcast, as the podcast discussion on gestational diabetes covered additional items beyond those initially prepared in the semistructured podcast guide.

The knowledge questionnaires for each of these podcasts will address 3 key themes: risk factors, symptoms, and services or treatments. Examples of the questions asked can be found in Table 1.

The only qualitative question asked at the end of each podcast is the following:

“Do you have any further comments on the podcast (duration or formatting), questions asked, or recommendations for future podcasts?”

**Table 1:** Example of questions asked on knowledge and awareness.

Topic	Area being assessed	Examples of question asked before and after the podcast
Gestational diabetes	Knowledge	Is obesity or BMI a risk factor for gestational diabetes mellitus?
Gestational diabetes	Awareness	Have you heard of metformin?
Mental health	Knowledge	Is a history of mental illness in a blood relative family member a risk factors for mental illness?
Mental health	Awareness	Are you aware of risk factors for mental health problems?

### Study Population

The study will collect data from undergraduate general nursing students from 4 universities located across Ireland, South Africa, and Malawi.

### Sampling and Sample Sizes

The sample will consist of a cohort of undergraduate nursing students from University College Cork (UCC) and the University of Galway (Ireland), the University of Fort Hare (South Africa), and Mzuzu University (Malawi). These students will be recruited through invitation via the university they are attending. Entry into the study will be entirely voluntary.

The main aim is to compare the pre- and postpodcast scores on knowledge and awareness differences with a paired 2-tailed *t* test. The inputs that will be used are the expected mean (SD) of the paired differences. As this is a feasibility study, a formal sample size calculation is not required [28].

### Inclusion and Exclusion Criteria

Exclusion criteria include those who are unable to complete the web-based questionnaires due to a lack of internet connection. These surveys are solely available via Google Forms and are thus inaccessible without an internet connection. Participants will be excluded if they are not a current resident and a registered undergraduate general nursing student of Ireland, South Africa, or Malawi and do not agree to consent to the study. As this is a feasibility study, we are targeting a small cohort of individuals who are convenient to the research team. Inclusion criteria encompass those aged 18 years and older.

### Data Collection and Management

Questionnaires will be made using Google Forms, which are stored on UCC’s Microsoft OneDrive. The questionnaires will include closed-ended questions using a point scale, with points given to the tick-the-box answers of “Yes”, “No,” or “I don’t know.” These questionnaires will be distributed to UCC (Ireland), University of Galway (Ireland), University of Fort Hare (South Africa), and Mzuzu University (Malawi). Regarding the data collection, the investigators will not seek names or emails from the participants, and the data will be fully anonymized. Fully anonymized data are achievable in this study, as participants are not required to complete two different surveys. The pre- and postpodcast questions are on a single Google Form, separated only by the embedded podcast file (not 2 separate survey forms), allowing a direct comparison of survey responses without requiring participant contact details. Once the survey is submitted, the option to withdraw will no longer be available. Answers to our survey (questionnaires) will be stored on UCC-supplied Google Forms and uploaded to OneDrive. Data on knowledge and awareness will be collected from both pre- and postpodcast surveys. Information on gender, age, year of study, and the university the student is attending will also be stored, along with the consent forms. The UCC-supplied OneDrive will also be used during data collection and data analyses due to the ability to store data generated on the cloud automatically. Data will be stored for a minimum of 10 years. The data will be controlled by the UCC study members. General Data Protection Regulation will be observed at all times with all information anonymized.

Data collection tools will be structured according to the research themes outlined as follows:

#### Podcast Knowledge

Participants will be asked about their knowledge of the global health topic by assessing knowledge of symptoms, risk factors, and treatment options associated with the disease. The Google Forms will present an individual question on symptoms, risk factors, and treatments and ask respondents to indicate whether they are symptoms, risk factors, and treatment options of the condition with response options “Yes,” “No,” or “I Don't know.” For the prompted symptoms items, “No” and “I Don't Know” responses will be combined and scored 0, and “Yes” responses will be scored 1. Total scores for prompted awareness of warning signs and risk factors as well as knowledge of symptoms, risk factors, treatment options, government policies, or screening programs will be calculated by adding the recorded responses together.

#### Podcast Awareness

To achieve this objective, terms on awareness will focus on whether participants have previously heard of terminology associated with the specific podcast and are aware of services available for that health topic or whether they have heard of the condition or disease. With regard to awareness assessment, a scoring system will be implemented. For each question item, “No,” and “I Don't Know” responses will be combined and scored 0, and “Yes” responses will be scored 1.

### Data Analysis

Microsoft Excel and Google Forms will be used to capture raw quantitative data and to execute the descriptive statistics in the form of percentages and frequencies. Descriptive statistics, including percentages, means, and standard deviations will be calculated for participants’ survey responses. For the pre- and posttest analysis, a paired 2-tailed *t* test analysis for the questions that assess medical knowledge will be performed. The means of both the pre and posttests will be calculated. The pretest will act as the control, and therefore, *P* values will be determined to identify if the results are statistically significant. This analysis will enable the observation of whether the podcasts were an effective intervention tool by examining a higher posttest mean score. Significance will be defined as *P*<.05.

This is an international feasibility study, and it would be of great interest to analyze participants’ country of residence in conjunction with the level of knowledge and awareness gained from the podcast to give us an insight into which country would benefit most from this form of audio learning. To conduct this, we will perform a subgroup analysis in SPSS. Furthermore, men and women have been shown to learn differently, with women preferentially being auditory learners [29]. Therefore, a subgroup analysis on gender will be carried out on SPSS. This research will help to clarify whether podcasting is a useful educational resource that is of benefit to both sexes as an alternative source of learning [30].

For this study, we only have 1 open-ended question, which is “Do you have any further comments on the podcast (duration or formatting), questions asked, or recommendations for future podcasts?” We will conduct a thematic analysis of the responses to open-ended questions [31]. To analyze and describe the comments, a coding frame will be developed. If responses contain 2 or more different statements, these statements or sentences will be coded separately or line by line.

### Ethical Considerations

The study received ethical approval from the UCC’s Social Research Ethics Committee (ID 2022-027A1). The approval obtained from UCC sufficed as ethics coverage for the University of Galway in Ireland. Ethics approval was also received by the Mzuzu University Research Ethics Committee (ID MZUNIREC/DOR/23/28) and by the Inter-Faculty Research Ethics Committee at the University of Fort Hare (ID CIL002-21) in South Africa. The study will conform with the precepts of the Declaration of Helsinki on Human Rights of 1975. This research will involve participation through remotely answering survey questions and listening to podcasts. The survey is available in the English language only, as it is the official language in Ireland, Malawi, and South Africa. Participation in this research is not likely to cause any harm or pose a risk to the study participants’ well-being.

Informed consent will be explicitly sought from the study participants at the beginning of the survey, and participants are required to be 18 years of age and older. Only undergraduate nurses can take part in this research. Participate confidentiality will be upheld throughout the study process. To maintain confidentiality, no emails, names, or contact information will be required.

The study data will be stored on a password-protected laptop to which only the research team will have access. Participation in this study is entirely voluntary, and participants retain the right to withdraw their participation and data at any time while filling out the survey. However, once the survey is submitted, participants cannot withdraw because the survey is anonymous, and we can therefore not identify who completed the survey. Participants will not receive remuneration or compensation for taking part in the study.

## Results

Data collection is currently being conducted and will be carried out until February 2024. The quantitative and qualitative data are expected to be analyzed by March 2024.

## Discussion

### Comparison With Prior Work

This study, to the best of our knowledge, is the first podcast intervention study to compare the impact of podcasting on nursing education internationally. This is extremely pertinent considering the changing landscape of third-level education, the impact of web learning, and the shift away from conventional approaches [32]. This study may highlight the idea that the next generation may be keener on using this technology, as they have grown up with e-learning and internet-based content.

The implementation of podcasting in nursing education is a strategy that has been considered in previous literature. O'Connor et al [33] (2020) identified that the next generation of nursing students has grown up with technology and thus are mostly learners with diverse digital literacy abilities and perhaps a need for interactive, learner-centred courses. Fortunately, podcasting is emerging as a viable educational option. Abate [34] (2013) demonstrated that when undergraduate nursing students listened to a segmented podcast, they had increased scores on multiple choice exams and case study tests, compared to face-to-face lectures and unsegmented podcasts. Likewise, Burke and Cody [35] (2014) reported that 86% of undergraduate nursing students felt that podcasts enriched their learning, and an astonishing 94% of these students would recommend podcasting for other courses.

### Expected Findings

This study will investigate the impact of informational podcasts on the attainment of knowledge and awareness in undergraduate nursing students. It will provide valuable insights into the impact of podcasts in education settings across different countries. We anticipate that results from this study could illuminate whether educational podcasts serve as an impactful learning resource for undergraduate nursing students.

### Future Research

Other populations apart from undergraduate general nursing students should be included in further studies to determine the appropriateness of this intervention across third-level education. If the results are positive, this feasibility study will be used to inform future research on podcasting in third-level education.

### Strengths and Limitations

The role of social media platforms as a medium to deliver education has been under development for several years. This research will provide a clear contribution focusing on the impact of one such platform—YouTube—for undergraduate nursing students in the areas of knowledge and awareness on a set of given topics. This research is international and will enable a greater understanding of the usefulness of podcasts in a high-income country (Ireland) compared to middle-to-low-income countries (South Africa and Malawi). Nonresponse is one of the limitations of this study. Pre- and postpodcast questionnaires are planned to be administered before and after listening to a podcast recording. This may cause participants to become mentally fatigued and not fill out the questionnaire following the podcast, resulting in a reduced response rate. Another limitation of this study is selection bias. For this study, only general undergraduate nursing students will be used to gather information, and thus, they could skew the results due to the level of knowledge they should already have from their undergraduate studies. The generalisability of the study may be limited by the cohort only being composed of nursing students.

### Conclusions

Results from this study will allow for an investigation into the impact of podcasts in different settings, a high-income country (Ireland), an upper-middle–income country (South Africa), and a low-to-middle–income country (Malawi). The data gathered from this feasibility study will provide more clarity on the potential utility of podcasts as an intervention tool. We will gather data regarding listener demographics (ie, country of residence, age, gender, and year of study) to ascertain the outreach of the podcasts, which could shape future studies on podcasts as an educational tool. If the results of our study are positive in terms of an increase in knowledge and awareness, we hope to identify other populations that might benefit from this sort of educational resource.

## Abbreviations

UCC: University College Cork
WHO: World Health Organization

## Data Availability

The media data that support the methodology of this study are openly available on Figshare [36]. This is covered by the Creative Commons Attribution license (CC BY 4.0 license).

## References

[1] Hammersley Audible revolution. The Guardian.

[2] Colombo G, Curtis F Jr (2018). Absolute Beginner's Guide to Podcasting.

[3] Rime J, Pike C, Collins T (2022). What is a podcast? Considering innovations in podcasting through the six-tensions framework. Convergence.

[4] Cho D, Cosimini M, Espinoza J (2017). Podcasting in medical education: a review of the literature. Korean J Med Educ.

[5] Brew-Girard E, Brown R, Salter E, Hattersley C, Hodge O, Leonard X, Macdonald K, Mupanemunda G, Quinn M, Rahman J, Roberts A, Skuse K, Tran M, De Souza S (2023). Hunting for Pearls: A Qualitative Analysis of the Reflections of Students Creating Psychiatric Podcasts. AMEP.

[6] Chin A, Helman A, Chan T (2017). Podcast use in undergraduate medical education. Cureus.

[7] Mitchell G, Scott J, Carter G, Wilson CB (2021). Evaluation of a delirium awareness podcast for undergraduate nursing students in Northern Ireland: a pre−/post-test study. BMC Nurs.

[8] Crooks S, Stark P, Carlisle S, McMullan J, Copeland S, Wong WYA, Blake D, Lyons E, Campbell N, Carter G, Wilson CB, Mitchell G (2023). Evaluation of a co-designed Parkinson's awareness audio podcast for undergraduate nursing students in Northern Ireland. BMC Nurs.

[9] O'Connor S, Daly CS, MacArthur J, Borglin G, Booth RG (2020). Podcasting in nursing and midwifery education: an integrative review. Nurse Educ Pract.

[10] Reuters Institute Digital News Report 2020.

[11] Knowledge. Meriam-Webster.

[12] Merriam-Webster. Awareness.

[13] Maternal mortality. World Health Organization.

[14] Diabetes. World Health Organization.

[15] Malanda B (2017). Lessons from WINGS. Diabetes Res Clin Pract.

[16] Guariguata L, Linnenkamp U, Beagley J, Whiting D, Cho N (2014). Global estimates of the prevalence of hyperglycaemia in pregnancy. Diabetes Res Clin Pract.

[17] Hod M, Kapur A, Sacks DA, Hadar E, Agarwal M, Di Renzo GC, Roura LC, McIntyre HD, Morris JL, Divakar H (2015). The International Federation of Gynecology and Obstetrics (FIGO) Initiative on gestational diabetes mellitus: a pragmatic guide for diagnosis, management, and care. Int J Gynaecol Obstet.

[18] Data on the size of the HIV epidemic. World Health Organization.

[19] Sung H, Ferlay J, Siegel RL, Laversanne M, Soerjomataram I, Jemal A, Bray F (2021). Global cancer statistics 2020: GLOBOCAN estimates of incidence and mortality worldwide for 36 cancers in 185 countries. CA Cancer J Clin.

[20] Cervical cancer. World Health Organization.

[21] Malnutrition. World Health Organization.

[22] The 17 goals, sustainable development. United Nations.

[23] Mental health. World Health Organization.

[24] Arias D, Saxena S, Verguet S (2022). Quantifying the global burden of mental disorders and their economic value. EClinicalMedicine.

[25] Bowen DJ, Kreuter M, Spring B, Cofta-Woerpel L, Linnan L, Weiner D, Bakken S, Kaplan CP, Squiers L, Fabrizio C, Fernandez M (2009). How we design feasibility studies. Am J Prev Med.

[26] Google forms: sign-in. Google.

[27] Lucero‐Prisno DE, Shomuyiwa DO, Kouwenhoven MBN, Dorji T, Odey GO, Miranda AV, Ogunkola IO, Adebisi YA, Huang J, Xu L, Obnial JC, Huda A, Thepanondh S, Dayrit MM, Evardone SB, Lamawansa MD, Solomon Ethiopia S, Aziato L, Adongo PB, Samai MH, Garcia FB, Villaruz JF, Karunathilake IM, Li H, Azanza PA, Findlay I, Wong MCS (2023). Top 10 public health challenges to track in 2023: shifting focus beyond a global pandemic. Public Health Chall.

[28] Sluggett JK, Page AT, Chen EYH, Ilomäki Jenni, Corlis M, Van Emden J, Hogan M, Caporale T, Angley M, Hilmer SN, Ooi CE, Bell JS (2019). Protocol for a non-randomised pilot and feasibility study evaluating a multicomponent intervention to simplify medication regimens for people receiving community-based home care services. BMJ Open.

[29] Wehrwein EA, Lujan HL, DiCarlo SE (2007). Gender differences in learning style preferences among undergraduate physiology students. Adv Physiol Educ.

[30] Baharu H (2019). Male and Female EFL Students’ Learning Style at Muhammadiyah University of Makassar. ELTWW.

[31] Braun V, Clarke V (2006). Using thematic analysis in psychology. Qual Res Psychol.

[32] Wongwatkit C, Srisawasdi N, Hwang G, Panjaburee P (2016). Influence of an integrated learning diagnosis and formative assessment-based personalized web learning approach on students learning performances and perceptions. Interact Learn Environ.

[33] O'Connor S, Daly CS, MacArthur J, Borglin G, Booth RG (2020). Podcasting in nursing and midwifery education: an integrative review. Nurse Educ Pract.

[34] Abate KS (2013). The effect of podcast lectures on nursing students’ knowledge retention and application. Nurs Educ Perspect.

[35] Burke S, Cody W (2014). Podcasting in undergraduate nursing programs. Nurse Educ.

[36] An international study evaluating the appropriateness of podcasts to improve the knowledge and awareness of selected health topics amongst undergraduate general nursing students: protocol for a feasibility study. Figshare.

